# Melissopalynological Characterization of North Algerian Honeys

**DOI:** 10.3390/foods2010083

**Published:** 2013-03-07

**Authors:** Samira Nair, Boumedienne Meddah, Abdelkader Aoues

**Affiliations:** 1 Laboratory Research on Biological Systems and Geomatics, Faculty of Nature and Life, University of Mascara, 29000, Mascara, Algeria; E-Mail: meddah19@yahoo.fr; 2 Laboratory of Experimental Biotoxicology, Biodepollution and Phytoremediation, University of Es-Senia, 31000, Oran, Algeria; E-Mail: aoues_a@yahoo.fr

**Keywords:** pollen analysis, Algerian honey, cluster analysis, pollen spectrum, botanical origin

## Abstract

A pollen analysis of Algerian honey was conducted on a total of 10 honey samples. The samples were prepared using the methodology described by Louveaux *et al*., that was then further adapted by Ohe *et al*. The samples were subsequently observed using light microscopy. A total of 36 pollen taxa were discovered and could be identified in the analyzed honey samples. Seventy percent of the studied samples belonged to the group ofmonofloral honeys represented by *Eucalyptus globulus*, *Thymus vulgaris*, *Citrus* sp. and *Lavandula angustifolia*. Multifloral honeys comprised 30% of the honey samples, with pollen grains of *Lavandula stoechas* (28.49%) standing out as the most prevalent. Based on cluster analysis, two different groups of honey were observed according to different pollen types found in the samples. The identified pollen spectrum of honey confirmed their botanical origin.

## 1. Introduction

Honey, apart from a variety of stable elements, contains floral pollen grains that are collected by honeybees along with the nectar. The presence of the floral pollen grains affords the possibility of identifying the botanical and geographical origin of honey [1].

Diversity of vegetation in Algeria makes possible diversification of honey production. The apiculture in Algeria is widely practiced in the mountainous region (Kabylie, Aurès mountains), in the coastal plains (Mitidja), in the interior plains (Mascara) and in the valleys of large wadis (Soummam), the deserted areas of the highlands and the southern region of the country being the only exceptions to honey production [2]. The botanical origin of honey is one of the most important parameters of honey quality [3,4,5]. The quality of honey depends on the melliferous plants that bees use in their nourishment [6]. The nectar obtained from different melliferous plants has different characteristics and applications, both in medicine and in the food industry [4].

Taxa of pollen are used to indicate the floral nectar sources utilized by bees to produce honey [7,8,9,10] and are also an essential tool to indicate the geographic origin of the honey [11,12].

The aim of this work was to provide information on the geographical and botanical origin of the honey samples collected and to identify the nectar sources foraged by the bees.

## 2. Materials and Methods

### 2.1. Sample Collection

Ten samples of honeys produced in two regions of northern Algeria were collected from beekeepers in 2006. The samples were stored in a refrigerator in airtight plastic containers until analysis; these samples were of different floral types (unifloral and multifloral honey).

### 2.2. Pollen Analysis

The melissopalynological characteristics, both qualitative and quantitative, were studied following Louveaux *et al.* [13] and Ohe *et al.* [14]. Ten grams of honey were dissolved in 20 mL of distilled water. This mixture was divided into two centrifuge tubes of 15 mL, and centrifuged for about 5 min; at 3000 tr/min. Distilled water was again added to the sediment, repeating the previous operation. Approximately 5 mL of glycerine-water 1:1 were added to the sediment, and it was left to rest for 30 min. After this time, the sample was centrifuged. The sediment was removed with the aid of a stylet, embedded in glycerine jelly and deposited on a microscopic slide, sealing with paraffin wax. The identification of each pollen grain in the treated honey sample was performed with the aid of a pollen atlas [15], and other published floras [16,17,18,19], which gave brief descriptions of the observed pollen in the samples.

The frequency classes of pollen grains are given as predominant pollen (>45%), secondary pollen (16%–45%), important minor pollen (3%–15%) and minor pollen (1%–3%) [13].

### 2.3. Statistical Analysis

To classify the samples, a cluster analysis was applied. This analysis can classify the data in groups of similarity (clusters) [20].

Cluster analysis is comprised of a series of multivariate methods that are used to find true groups of data or stations. In clustering, the objects are grouped such that similar objects fall into the same class.

Cluster analysis was displayed in order to find similarities between the honey samples. In order to do so, Euclidean’s hierarchical cluster method for pattern recognition was used.

## 3. Results and Discussion

**Figure 1:**
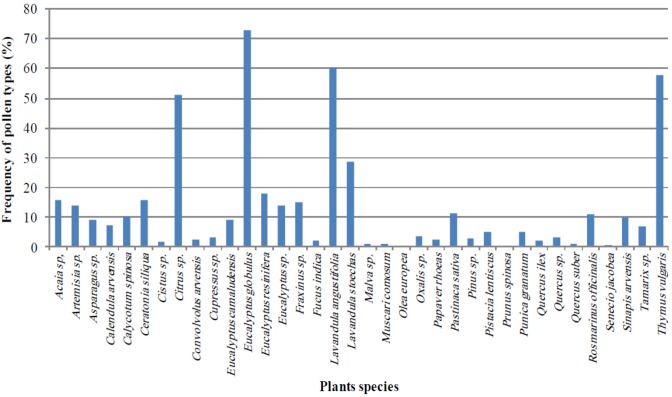
Somatorium of the pollen frequency of the identified plant species in ten samples of Algerian honeys.

The result of 10 studied samples in this work shows that only four pollen types presented frequency higher than 45%; the main botanical species for honey production in Algeria are found to be *Eucalyptus globulus*, *Thymus vulgaris*, *Citrus* sp. and *Lavandula angustifolia*, (Figure 1).

Honey sample 1, from the locality Ménaouer, contained pollen grains from *Thymus vulgaris* at a percentage of nearly 58%; thus, the pollen of this species is dominant, suggesting that this plant is the chief source of pollen and nectar in bee foraging.

Honey sample 5, from the locality Freguig, has *Lavandula angustifolia* as the predominant pollen. Of the ten samples, three (S8, S9 and S10) presented very high percentages of *Eucalyptus globulus*; in two samples (S2 and S7), *Citrus* was predominant (Table 1).

**Table 1:** Pollen content in the honey samples (%).

Pollen Taxa/Family	Samples									
	Meneour	Guetna	Makda	Hacine	Freguig	Ain fares	Bouguirat	Sidi ali	Sirat	Hadjadj
	(S1)	(S2)	(S3)	(S4)	(S5)	(S6)	(S7)	(S8)	(S9)	(S10)
*Acaia* sp. (*Mimosaceae*)									15.6	
*Artemisia* sp. (*Composeae*)		14								
*Asparagus* sp. (*Liliaceae*)			9.37			8.99				
*Calendula arvensis* (*Composeae*)				7.02	7.5					
*Calycotum spinosa* (*Papilionaceae*)					10.1					
*Ceratonia siliqua* (*Fabaceae*)			15.62							
*Cistus* sp. (*Cistaceae*)			1.5							
*Citrus* sp. (*Rutaceae*)		46		45			62.5			
*Convolvolus arvensis* (*Convolvulaceae*)										2.5
*Cupressus* sp. (*Cupressaceae*)					3.08					
*Eucalyptus camaludensis* (*Myrtaceae*)							12	6.98	12	5
*Eucalyptus globulus* (*Myrtaceae*)								86.2	52.4	80.22
*Eucalyptus resinifera* (*Myrtaceae*)									18	
*Eucalyptus* sp. (*Myrtaceae*)	15			20		22.96	9.88	0.57		
*Fraxinus* sp. (*Oleaceae*)			14.84							
*Fucus indica* (*Cactaceae*)	1.98									
*Lavandula angustifolia* (*Lamiaceae*)					60.33					
*Lavandula stoechas* (*Lamiaceae*)		21				35.98				
*Malva* sp. (*Malvaceae*)	0.43			1.19						
*Muscari comosum* (*Liliaceae*)	0.78									
*Olea europea* (*Oleaceae*)							0.18			
*Oxalis* sp. (*Oxalidaceae*)		3.5								
*Papaver rhoeas* (*Papaveraceae*)				2.26	1.76					3.5
*Pastinaca sativa* (*Apiaceae*)	11.98			15	15			5.97		7.78
*Pinus* sp. (*Pinaceae*)		6.5	1.95					0.11		
*Pistacia lentiscus* (*Anacardiaceae)*	5.98				9.26			0.14		
*Prunus spinosa* (*Rosaceae*)	4.44									
*Punica granatum* (*Pinucaceae*)							5			
*Quercus ilex* (*Fagaceae*)					2.05					
*Quercus* sp. (*Fagaceae*)						3.02				
*Quercus suber* (*Fagaceae*)					1.02					
*Rosmarinus officinalis* (*Lamiaceae*)						18.95			2.51	
*Senecio jacobea* (*Asteraceae*)	0.59									
*Sinapis arvensis* (*Brassicaceae*)		9		9.52			10.44			
*Tamarix* sp. (*Tamaricaceae*)			7.03							
*Thymus vulgaris* (*Lamiaceae*)	57.91									

Quantitative analysis has shown low pollen concentrations in the studied honey samples, 5 samples belonged to the I class of representativity (under-represented honeys, with less than 20,000 pollen grains in 10 g honey), 5 to the II class (normal honeys, with 20,000 to 100,000 PG/10 g). Our results are quite in agreement with Ouchemoukh *et al.* [21]; these authors found in their study of 11 Algerian samples lower PG/10 g values, ranging from 20 × 103 until 40 × 103. Their samples were collected in various regions of the province Bejaia.

The results of a study by Makhloufi *et al*. [16] on the pollen richness of 66 Algerian honeys found the values for the PG/10 g for the classes I, II, III and V to be, respectively, 33%, 40.9%, 22.7% and 3%.

The results of qualitative pollen analysis indicate the diversity of resources utilized by honeybees in the region of investigation. The botanical families *Myrtaceae*, *Rutaceae* and *Lamiaceae* were most frequently found in the samples.

Out of 66 Algerian honeys analyzed by Makhloufi *et al.* [22], the main botanical species for Algerian honeys were found to be *Eucalyptus* sp., *Umbelliferae* (above all *Pimpinella*), *Hedysarum*, Cruciferae, Compositae (mainly *Carduus*), *Trifolium* sp. and, to a lesser extent, *Echium, Rubus* and *Citrus*.

**Figure 2:**
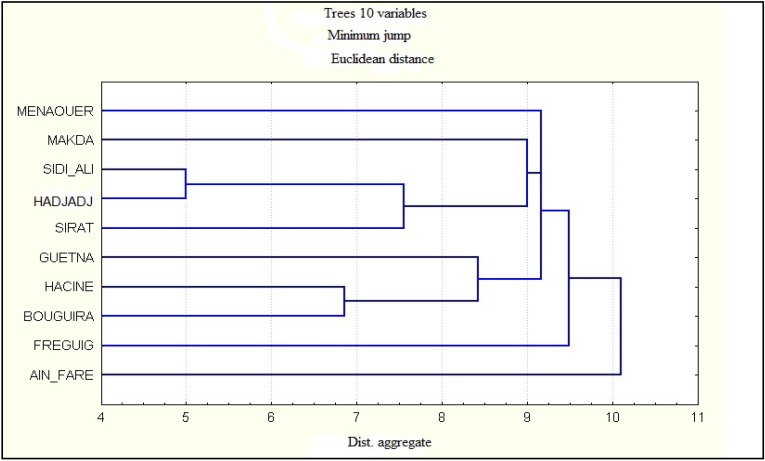
Dendrogram of cluster analysis.

With the dendogram that corresponds to the geographical origin of the honey samples, it was possible to distinguish two different groups (Figure 2). The first group is composed of honey produced in Ain fares; the second cluster clearly creates two separate subgroups. The first subgroup includes the stations Guetna, Hacine and Bouguirat, with *Citrus* sp. being the most common plant species pollen in the samples of these stations. The second includes the stations Sidi ali, Hadjadj and Sirat, a representative amount of *Eucalyptus globulus* occurred in these samples. The botanical composition of regional honey depends on the climatic conditions during the apicultural period.

## 4. Conclusion

From the 10 studied samples, a total of 36 taxa of nectariferous honey plants were identified. The specific richness of the investigated honey varied from 1 to 9 taxa. The melissopalynological analyses showed that the most dominating pollens in honey samples were *Myrtaceae*, *Rutaceae* and *Lamiaceae*. Cluster analysis distinguishes two different groups of Algerian honey.
